# Probing the impact of temperature on molecular events in a developmental system

**DOI:** 10.1038/srep13124

**Published:** 2015-08-19

**Authors:** David Cheung, Jun Ma

**Affiliations:** 1 Division of Biomedical Informatics, Cincinnati Children’s Research Foundation, 3333 Burnet Avenue, Cincinnati, OH 45229, USA; 2 Division of Developmental Biology, Cincinnati Children’s Research Foundation, 3333 Burnet Avenue, Cincinnati, OH 45229, USA

## Abstract

A well-appreciated general feature of development is the ability to achieve a normal outcome despite the inevitable variability at molecular, genetic, or environmental levels. But it is not well understood how changes in a global factor such as temperature bring about specific challenges to a developmental system in molecular terms. Here we address this question using early *Drosophila* embryos where the maternal gradient Bicoid (Bcd) instructs anterior-patterning (AP) patterning. We show that temperature can impact the amplitude of the Bcd gradient in the embryo. To evaluate how molecular decisions are made at different temperatures, we quantify Bcd concentrations and the expression of its target gene *hunchback* (*hb*) in individual embryos. Our results suggest a relatively robust Bcd concentration threshold in inducing *hb* transcription within a temperature range. Our results also reveal a complex nature of the effects of temperature on the progressions of developmental and molecular events of the embryo. Our study thus advances the concept of developmental robustness by quantitatively elaborating specific features and challenges—imposed by changes in temperature—that an embryo must resolve.

Temperature is an environmental factor that impacts almost all biochemical reactions inside a cell. For developmental systems such as *Drosophila* embryogenesis, temperature fluctuations pose a significant challenge to the orderly morphological progression that depends on coordinated cellular decisions both spatially and temporally. This challenge may be appreciated by considering further the fact that temperature can alter the “rate” of development^1^,^2^,^3^,^4^,^5^,^6^. Consider two embryos that were developing at two different temperatures and have now reached the stage of hatching. Each of these embryos must have made countless cellular and molecular decisions and crossed numerous morphological landmarks to reach this “final” stage despite a significant difference in how long it has taken them at each and every step.

From the perspective of developmental biology, the hypothetical consideration of these two embryos simply provides an illustration of the well-appreciated robust nature of developmental systems^7^,^8^,^9^,^10^,^11^,^12^,^13^. But it remains largely unknown, from a biochemical perspective, how individual molecular decisions or events that control developmental processes are impacted by temperature. While the effects of temperature on a biochemical reaction can be systematically and experimentally studied in purified or reconstituted *in vitro* systems, the complexity of developmental systems and the interwoven and robust nature of regulatory networks controlling developmental processes will grossly complicate similar considerations^1^. Furthermore, molecular decisions and events in developmental systems may have time-constraints imposed by, or may play a leading role in driving forward, the irreversible morphological progression.

To fully appreciate and better understand the concept of developmental robustness, it is necessary to gain knowledge—however incomplete or limited in scope it might be—about the impact of temperature on molecular events relevant to developmental decisions. Here we approach this problem using a developmental system at a time involving relatively “few” components and relatively “simple” regulatory relationships. In particular, we use the patterning system along the anterior-posterior (AP) axis of the early *Drosophila* embryo. Here the initial regulatory input controlling AP patterning is derived from the maternal gene *bicoid* (*bcd*), which encodes a morphogenetic protein forming a concentration gradient along the AP axis^14^,^15^,^16^,^17^,^18^,^19^.

Recent studies suggest that, under the conditions when temperature is not a variable, Bcd is a primary activator input for the zygotic expression of the gap gene *hunchback* (*hb*) (ref. 14,18,20, 21, 22, 23, 24, 25, 26). However, it has been suggested that changes in temperature can cause the *hb* expression boundary to become unrelated to Bcd input properties in the embryo^9^. But this suggestion was based on data extracted from embryos that had considerable age uncertainties, leaving it an open question as to how temperature affects molecular decisions and events in the early *Drosophila* embryo. Specifically, does temperature affect the amplitude or the exponential shape of the Bcd gradient, or both? The earlier study suggested that temperature has a significant impact on the Bcd gradient shape^9^ but how it impacts the amplitude has not been well resolved experimentally. In addition, does temperature affect the developmental system in such a way that would make local Bcd concentration largely irrelevant to *hb* boundary formation as previously suggested^9^, or are some aspects of the Bcd-*hb* relationship relatively robust to changes in temperature?

To address these and other questions, we perform quantitative measurements in embryos maturing at different temperatures. In our study we made specific considerations to screen embryos for their developmental age and enhance the reliability of data relevant to the questions that we address. Our results show that the amplitude of the Bcd concentration gradient exhibits a higher degree of sensitivity to temperature than its length constant (i.e., the exponential shape). Our results also show that certain aspects of the relationship between Bcd and *hb*, e.g., threshold-dependent transcriptional activation, are maintained (but only to a degree) in embryos maturing at different temperatures. These new findings contrast with previous suggestions^9^. In addition, we present results that support a complex nature of the impact of temperature on a developmental system even at a time when molecular decisions are governed by “simple” regulatory relationships. They underscore the value of the technical considerations that we have made in our current study, particularly with respect to developmental age screening and the generation of experimental data in embryos on a side-by-side basis to permit direct and meaningful comparisons. Together, our study documents specific challenges an embryo must face at its earliest stages in coping with the impact of temperature, thus elaborating the concept of developmental robustness in specific and molecular terms.

## Results

### Experimental design

**Figure 1:**
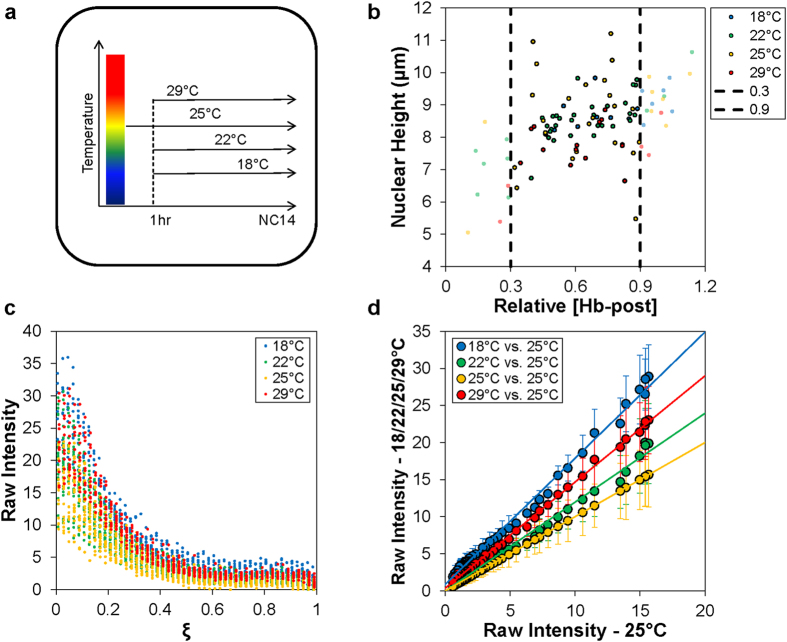
Experimental design and Bcd intensity measurements. (**a**) Shown is a graphical representation of the temperature maturation scheme used to generate the embryos for subsequent experiments. (**b**) Shown is a plot of the nuclear height measurements over the respective posterior *hb* expression level (relative to the anterior level) of individual embryos selected for further quantitative analysis. (**c**) Shown are raw fluorescent intensities detecting Bcd in individual embryos plotted as a function of fractional embryo length (*ξ*). For presentation purposes, data for different thermal cohorts are shown with 0.002 offsets on *ξ* axis to enhance visual clarity. (**d**) Shown are scatter plots of the mean raw Bcd intensity values from embryos maturing at 18 °C, 22 °C, 25 °C and 29 °C plotted against the intensities from embryos maturing at 25 °C. A linear fit is shown for each pairwise plot; error bars are standard deviation (s.d.).

To investigate how the environmental factor of temperature may affect the initial molecular events and properties of embryonic patterning along the AP axis, we performed quantitative measurements in whole mount embryos that matured at different temperatures. Given the complexity of the problem in hand, we made the following specific considerations in our experimental design and data analysis. First, to evaluate specifically the impact of temperature on molecular events that take place solely in the embryo, irrespective of those in the mother, we devised an experimental scheme that is shown in Fig. 1a. Here, 0–1 hr eggs were collected from wild type (*w*^1118^) females that were reared at 25 °C. These eggs/embryos were subsequently allowed to further mature at different temperatures (18 °C, 22 °C, 25 °C and 29 °C) until reaching early nuclear cycle (nc) 14 (see Methods and below). Second, to generate quantitative data suitable for evaluating the relationship between Bcd and *hb* expression properties in individual embryos maturing at different temperatures, we performed whole mount co-staining experiments combining immuno-staining to detect nuclear Bcd and FISH to detect cytoplasmic *hb* mRNA. Unlike Hb protein which represents a “delayed” response to the Bcd input, *hb* mRNA provides a measurement of a transcriptional response. As documented previously^14^,^21^,^22^,^27^ and further discussed below, we used experimental and analytical procedures that preserve a linear relationship between intensities of the detected fluorescent signals and molecular concentrations. Third, to permit direct comparisons between embryos maturing at different temperatures, all the staining and imaging steps were performed on a side-by-side basis.

Since the rate of development is directly affected by temperature^1^,^4^,^5^,^28^, our evaluations of molecular events depended on the chosen time definitions and material availability. We made the following specific considerations in our study with regard to time. First, we sought to establish quantitative comparisons of the molecular features in embryos of a comparable developmental age, as opposed to the chronological age (i.e., the absolute time after egg deposition). We used quantifiable morphological markers to estimate the developmental age of an embryo (see below). Nc14 is a critical developmental stage during which the Bcd gradient has been well established and it regulates the expression of its target genes such as *hb* (ref. 14,15,22,23,26,29, 30, 31, 32, 33, 34). It is also the stage when *hb* mRNA has accumulated to levels that allow reliable quantitative measurements 22,35,36. Second, we used the nuclear height, which is a well-documented and sensitive measure of time at 25 °C (ref. 29,37, 38, 39), as a common morphological standard for estimating the developmental ages of embryos maturing at different temperatures. The embryos chosen for our analysis were at early nc14 (see Fig. 1b), with an estimated 25 °C age equivalent not exceeding ~20 min into the interphase (see Methods). Third, in addition to this morphological standard, we used the posterior *hb* expression level (relative to the anterior level) as a molecular standard in a secondary screening for embryos used in quantitative evaluations. The ratio of posterior-to-anterior *hb* mRNA levels is a well-documented and reliable molecular indicator of developmental time at 25 °C (ref. 14,29), and we selected embryos within the range of 0.3–0.9 for further analyses (see Fig. 1b for a scatter plot of nuclear height (μm) and relative posterior *hb* level for embryos undergoing the secondary screening).

### Maturation temperature has a measurable impact on Bcd gradient properties in embryos

**Figure 2:**
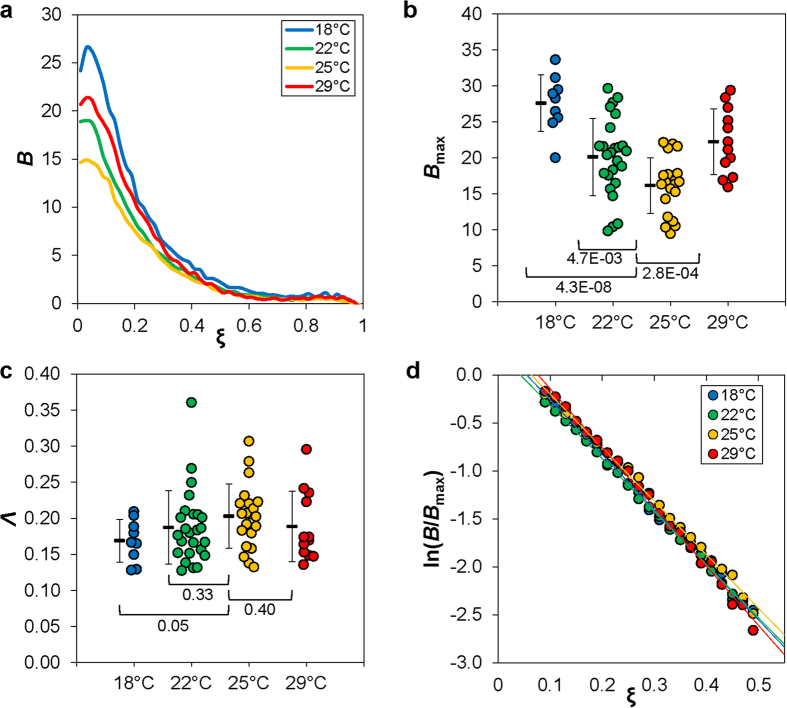
Properties of Bcd gradient profiles impacted by temperature. (**a**) Shown are mean Bcd intensities as a function of relative AP position *ξ*. Thermal cohorts are color coded: 18°C, 22 °C, 25 °C and 29 °C in blue, green, yellow, and red, respectively. (**b**) Shown is a swarm plot of the maximal Bcd intensities of individual embryos, with mean and s.d. shown alongside. Also shown are *p*-values from Student’s *t* tests for comparisons with measurements at the “normal” temperature of 25 °C. (**c**) Shown is a swarm plot of the calculated length constant values, *Λ* = *λ*/*L*, from individual embryos of the indicated thermal cohorts. d) Shown are the mean Bcd intensity values of the indicated thermal cohorts, expressed as ln(*B*/*B*_max_) and plotted as a function of *ξ*. This provides a visual representation of an overall similarity of the slopes, which are the negative reciprocals of the length constants. The fitted *Λ* values for the mean Bcd gradient profiles are: 0.16, 0.16, 0.17 and 0.15 at 18 °C, 22 °C, 25 °C and 29 °C, respectively. The technical considerations in our current study, such as the double-screening for embryo age, allowed us to generate high quality data for quantitative evaluations with sample sizes comparable to our published studies^14^,^27^,^41^. In an alternative analysis, we used a Bcd intensity dataset with more embryos under a reduced stringency in embryo age screening (*n* = 14, 41, 32, and 25 at 18 °C, 22 °C, 25 °C and 29 °C, respectively). We obtained 〈*B*_max_〉 = 25.5 ± 5.6, 19.4 ± 5.0, 16.5 ± 4.1 and 21.9 ± 5.4, respectively, with an identical amplitude ranking as shown in panel b. Importantly, using intensity noise (s.d./mean) as a measure of data quality, we found that an increase in sample size *n* did not necessarily improve data quality. To the contrary, *B*_max_ intensity noise was higher in embryos maturing at 18 °C and 29 °C when sample size *n* and, concurrently, age heterogeneity were both increased (noise increased from 14.5% to 22% for 18 °C and from 20.6% to 24.7% for 29 °C). These results document that a larger sample size *n* does not necessarily translate into a more reliable dataset when evaluating a developmental system that is highly dynamic.

Figure 1c shows a scatter plot of raw Bcd intensity measurements against fractional embryo length, *ξ* = *x*/*L*, in embryos maturing at different temperatures (see Supplementary Fig. S1 online for raw intensity data plotted for each thermal cohort separately). A visual inspection of these raw data indicates that, in contrast to previous reports^9^, they exhibit an appreciable degree of overall similarity at this initial level of investigation. We attribute this contrast to technical differences between our current and previous studies, such as our side-by-side experimental and imaging steps and a double screening of embryos to narrow the window of their developmental ages (*n* = 9, 26, 21 and 12 for 18 °C, 22 °C, 25 °C and 29 °C, respectively; see Fig. 2 legend for a discussion about data quality and sample size). According to an evaluation described previously^40^, the overall similarity of the Bcd gradient profiles is also evident in scatter plots of mean Bcd intensity data between embryos maturing at different temperatures (Fig. 1d; see Supplementary Fig. S2 online for additional scatter plot data showing the relationship between Bcd intensities in embryos maturing at different temperatures).

To analyze our Bcd intensity data quantitatively and provide an enhanced visual representation of the potential effects of temperature on Bcd gradient formation, we plot profiles of the mean Bcd intensity 〈*B*〉 as a function of relative AP position, *ξ*, for each thermal cohort (Fig. 2a). Here, the Bcd intensity data were expressed as background-adjusted “raw” values without any further modifications (see Methods). Under our experimental conditions^14^,^27^, these intensity values are linearly related to nuclear Bcd concentrations (in arbitrary units; a.u.), thus preserving the biological meanings of both the amplitude and shape of the observed Bcd gradient profiles in embryos maturing at different temperatures. In this study, we use *B*_max_ or *B*_0_, which represents maximal Bcd intensity or the intensity at the anterior of an embryo, respectively, as a proxy for the amplitude of an experimentally observed Bcd gradient profile.

The results shown in Fig. 2a reveal visibly discernable differences among the mean Bcd profiles in the anterior of the embryos maturing at different temperatures (see also Fig. 1d). Quantitatively, 〈*B*_max_〉 = 27.6 ± 4.0, 20.1 ± 5.4, 16.1 ± 3.8 and 22.3 ± 4.6 (errors are standard deviations, s.d.) at 18 °C, 22 °C, 25 °C and 29 °C, respectively (Fig. 2b; see Fig. 2 legend for further information). In comparison with 〈*B*_max_〉 in embryos maturing at the “normal” temperature of 25 °C, 〈*B*_max_〉 obtained at each of the other three temperatures is statistically different (*p* = 4.3 × 10^−8^, 4.7 × 10^−3^, and 2.8 × 10^−4^ for 18 °C, 22 °C and 29 °C, respectively; Student’s *t* tests). We obtained similar results using 〈*B*_0_〉 to evaluate Bcd gradient profile’s amplitude. In particular, 〈*B*_0_〉 = 24.2 ± 4.7, 19.0 ± 5.6, 14.6 ± 4.0 and 20.7 ± 4.6 at 18 °C, 22 °C, 25 °C and 29 °C, respectively (*p* = 4.6 × 10^−6^, 5.0 × 10^−3^, and 4.3 × 10^−4^ for 18 °C, 22 °C and 29 °C, respectively, in comparisons with 25 °C). These results show that maturation temperature of the embryos can have a significant impact on the amplitude of the Bcd gradient.

### Evaluating the effect of temperature on Bcd gradient profile’s exponential shape characteristics

Despite the differences in the amplitude of the Bcd gradient profiles in embryos maturing at different temperatures, their overall descending nature as a function of AP position is well preserved (Figs 1d and 2a). The shape characteristics of an exponential gradient profile are encapsulated by its length constant *λ* and in our study we used *Λ* = *λ*/*L* for quantifying the shape of a Bcd gradient profile. To evaluate the potential effect of temperature on the Bcd gradient shape, we first fitted the Bcd intensity data from the region of *ξ*  = 0.1 to 0.5 of individual embryos to an exponential function to obtain the length constant *Λ*. This region was chosen for fitting to avoid both the anterior where the Bcd gradient profile is known to deviate from an exponential function^9^,^27^ and the posterior where measurement errors dominate^14^,^20^. Figure 2c shows the mean length constant values (and s.d.) for embryos maturing at different temperatures: 〈*Λ*〉 = 0.17 ± 0.03, 0.19 ± 0.05, 0.20 ± 0.04, and 0.18 ± 0.05 at 18 °C, 22 °C, 25 °C and 29 °C, respectively. The mean length constant values at 18 °C and 25 °C are significantly different (*p* = 0.046), but the values at 22 °C and 29 °C are not significantly different from that at 25 °C (*p* = 0.33 and 0.40 for 22 °C and 29 °C, respectively). Figure 2d shows a linear plot of the mean Bcd intensity values, ln〈*B*/*B*_max_〉, against AP position *ξ*, where the fitted slope provides a direct visualization of the mean profiles’ length constant estimations^23^,^27^,^41^.

Our results described thus far show that the length constant of the Bcd gradient profile 〈*Λ*〉 and its amplitude, 〈*B*_0_〉 or 〈*B*_max_〉, exhibit differential sensitivities to temperature. Under the framework of an idealized synthesis-diffusion-decay model (SDD), the length constant of a steady-state exponential concentration gradient is determined by the protein’s diffusivity and stability, and its amplitude is additionally sensitive to the protein’s production rate. We will discuss the implications of our findings with regard to how the molecular properties of Bcd gradient formation are impacted by temperature (Discussion).

### The mean *hb* expression boundary position exhibits an overall robustness to maturation temperature

**Figure 3:**
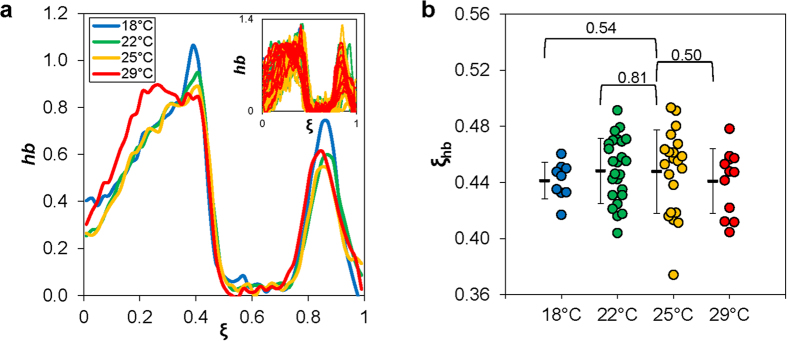
Evaluating the *hb* expression boundary in embryos maturing at different temperatures. (**a**) Shown are the mean *hb* expression profiles along the AP position *ξ* in embryos maturing at different temperatures (18 °C: blue, 22 °C: green, 25 °C: yellow, 29 °C: red). Inset shows data from individual embryos. The *hb* FISH intensity data shown here are background subtracted using a non-expressing region and subsequently normalized to peak levels (see Methods). (**b**) Shown are the measured *hb* expression boundary positions of individual embryos, with the mean and s.d. for the each thermal cohort shown. Also shown are *p*-values from Student’s *t* tests for comparisons with 25 °C.

To evaluate the potential effect of temperature on *hb* expression, we extracted *hb* mRNA FISH intensity data from individual embryos. Figure 3a (inset) shows normalized *hb* mRNA profiles of individual embryos as a function of *ξ*, with color coded according to maturation temperature (see Supplementary Fig. S3 online for each thermal cohort plotted separately). The mean *hb* expression profiles shown in Fig. 3a document a similarity in their boundary positions. To quantify the impact of temperature on *hb* expression boundary, we measured the boundary position of individual embryos, *ξ*_hb_, which is defined as the AP position at which the *hb* level reaches its half maximal. Figure 3b shows the mean values of such measurements: 〈*ξ*_hb_〉 = 0.44 ± 0.01, 0.45 ± 0.02, 0.45 ± 0.03, and 0.44 ± 0.02 at 18 °C, 22 °C, 25 °C and 29 °C, respectively. They document that 〈*ξ*_hb_〉 exhibits a relative insensitivity to maturation temperature (*p* = 0.54, 0.81 and 0.50 for 18 °C, 22 °C, and 29 °C, respectively, in comparisons with 25 °C).

### The relationship between Bcd and *hb* expression suggests a concentration-dependent action of Bcd

The measurable impact of temperature on Bcd gradient properties and a general robustness of the mean *hb* expression boundary position to temperature raised a question about whether and how temperature may affect the relationship between Bcd and *hb* expression. According to a previous study^9^, *hb* boundary formation is unrelated to Bcd gradient input properties across embryos that develop at different temperatures. As discussed in the Experimental Design section above, our co-staining procedure was designed to allow us to extract both Bcd intensities and *hb* mRNA FISH intensities from individual embryos, permitting evaluations of input-output relationships at different temperatures (see Supplementary Fig. S4 online for superimposed profiles of the mean Bcd intensities and mean *hb* FISH intensities extracted from embryos in each thermal cohort). It is useful to note that an input-output analysis effectively describes how the observed Bcd and *hb* properties are related to one another in embryos maturing at different temperatures given that these embryos are actually different in their chronological age (i.e., absolute time after egg deposition). It is based on an assumption that Bcd is the primary input for *hb* transcription in the anterior of the embryo at the chosen time, which is supported by the observed system behavior at 25 °C (ref. 14,20-23, 21, 22, 23,26,27,29,31,41). Thus our goal here is to extract quantitative information that can yield meaningful insights into how the earliest molecular decisions in AP patterning may be impacted by temperature (see also Discussion).

**Figure 4:**
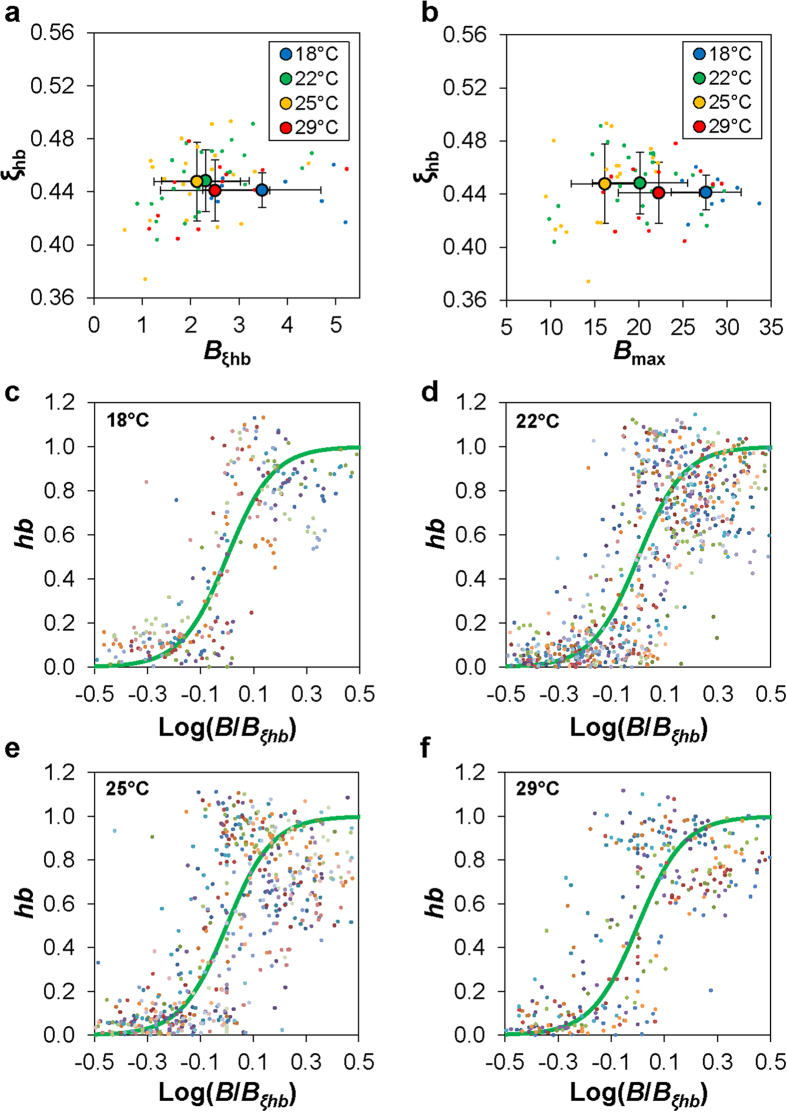
Concentration-dependent and cooperative nature of Bcd action in embryos. (**a**) Shown are scatter plots of the measured *hb* boundary position *ξ*_hb_ over the Bcd intensity at this position *B*_ξhb_ for individual embryos of the indicated thermal cohorts. Also shown are the mean and s.d. of each cohort. (**b**) Shown are scatter plots of the measured *hb* boundary position *ξ*_hb_ over the maximal Bcd intensity value *B*_max_ for individual embryos of the indicated thermal cohorts. Also shown are the mean and s.d. for each thermal cohort. (**c-f**) Shown are scatter plots of normalized *hb* intensity values over Bcd intensity for individual embryos of the indicated thermal cohorts. Here *B* is normalized to that at the *hb* boundary position (*B*_ξhb_) of the embryo and expressed on a log scale, and data are from the ξ range of 0.3 to 0.6 of individual embryos. The heavy green line in each panel shows a theoretical Hill function of *n* = 5.

To evaluate the input-output relationship between Bcd and *hb*, we first extracted from individual embryos the Bcd intensity value at the measured *hb* boundary position, *B*_ξhb_. In biochemical terms^42^,^43^,^44^,^45^,^46^, *B*_*ξ*hb_ represents an effective measure of the Bcd concentration required to bind to the *hb* enhancer to induce an on/off switch of *hb* transcription. We found that 〈*B*_*ξ*hb_〉 = 3.5 ± 1.2, 2.3 ± 0.90, 2.1 ± 0.90, and 2.5 ± 1.1 for embryos maturing at 18 °C, 22 °C, 25 °C and 29 °C, respectively (Fig. 4a). These results show that, except at 18 °C, 〈*B*_ξhb_〉 at either 22 °C or 29 °C is not significantly different from that at 25 °C (*p* = 2.1 × 10^−3^, 0.49, and 0.31 for 18 °C, 22 °C, and 29 °C, respectively, in comparisons with 25 °C). This result stands in contrast to the analysis of the relationship between *B*_max_ and *ξ*_hb_ in embryos maturing at different temperatures (Fig. 4b; see also Fig. 2b). Together they suggest that, except at 18 °C, the on/off switch of *hb* transcription (i.e., *hb* expression boundary formation) takes place at embryonic locations where the nuclear Bcd concentration crosses a common threshold at the tested temperatures.

To further evaluate the input-output relationship, we show scatter plots of normalized *hb* FISH intensities against normalized Bcd concentrations, log[*B*/*B*_*ξ*hb_], for embryos maturing at different temperatures (Fig. 4c–f). For illustrative purposes, each of these plots also displays a superimposed sigmoidal curve corresponding to a Hill function of *hb* = *B*^*n*^/[*B*^*n*^ + *B*_ξhb_^*n*^] with a coefficient of *n* = 5. These results provide a visual illustration that the relationship between Bcd and *hb* expression remains highly cooperative at each of the temperatures tested.

### The complex nature of the impact of temperature on molecular and developmental events

**Figure 5:**
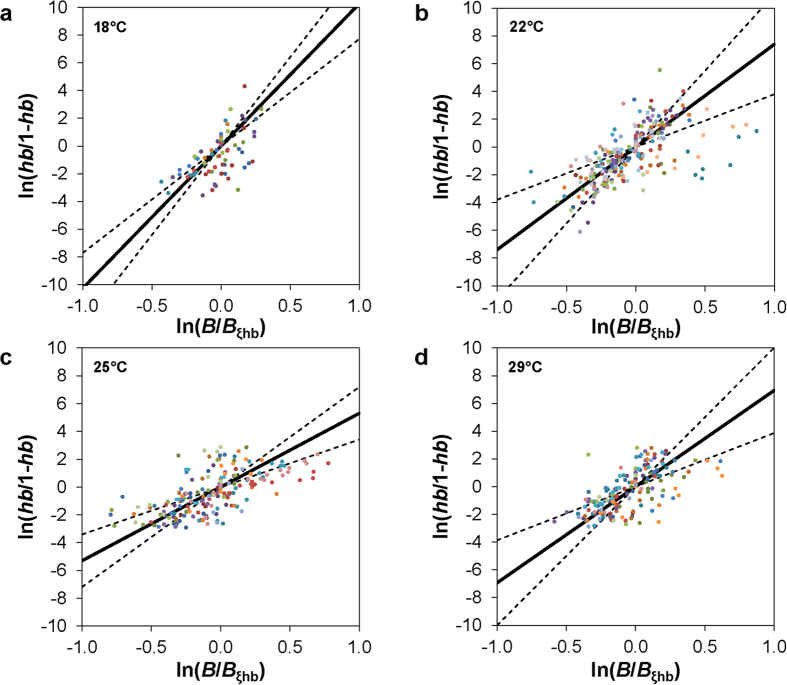
Estimation of Hill *n* values to account for the observed Bcd and *hb* properties in embryos. (**a-d**) Shown are linear plots to estimate Hill *n* values necessary to explain the Bcd-*hb* relationship in embryos of the indicated thermal cohorts. Data from individual embryos are color coded. The solid line and dotted lines in each panel represent the mean and s.d. of the fitted slopes obtained from individual embryos.

**Figure 6:**
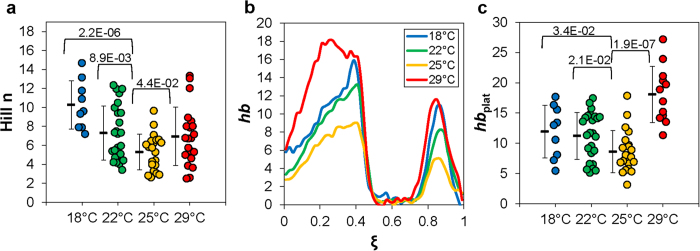
Temperature effects on calculated Hill *n* values and *hb* expression levels. (**a**) Shown is a swarm plot of the estimated individual Hill *n* values for individual embryos of the indicated thermal cohorts. These values are derived from linear fits shown in Fig. 5. (**b**) Shown are the mean, un-normalized *hb* expression profiles in embryos maturing at the indicated temperatures. (**c**) Shown are un-normalized levels of *hb* expression at the plateau region of individual embryos, with the mean and s.d. for each cohort shown. For panels a and c, *p*-values from Student’s *t* tests for comparisons with 25 °C are also shown.

To better quantify the Bcd-*hb* relationship and the potential impact of temperature, we fitted our data from individual embryo to the Hill function. We focused on the region of the embryo where the on/off switch of *hb* transcription takes place and used a linear fitting method in our data analysis^47^ (Fig. 5a–d and legend). Here the estimated Hill coefficient *n* for an embryo quantifies the degree of cooperative action necessary to explain the observed Bcd and *hb* properties assuming an input-output relationship. For embryos maturing at 18 °C, 22 °C, 25 °C and 29 °C, we obtained Hill 〈*n*〉 = 10.3 ± 2.6, 7.30 ± 2.9, 5.30 ± 1.9, 6.9 ± 3.1, respectively (Fig. 6a). These results further support a highly cooperative nature of the relationship between Bcd and *hb* expression at all of the temperatures tested. They also show that, under our experimental and analytical framework, the observed Hill 〈*n*〉 is not insensitive to temperatures (*p* = 2.2 × 10^−6^, 8.9 × 10^−3^, and 4.4 × 10^−2^ for 18 °C, 22 °C, and 29 °C, respectively, in comparisons with 25 °C). These results suggest that either the molecular interactions involved in Bcd-dependent *hb* transcription or the temporal relationships among the various molecular and developmental events (or both) are sensitive to temperature (see Discussion for further details).

The observed divergences of the calculated Hill 〈*n*〉 in embryos maturing at different temperatures suggest a complex nature of the impact of temperature on molecular and developmental events. They also raised a question of whether *hb* expression properties might exhibit additional differences in embryos maturing at different temperatures despite a similarity in 〈*ξ*_hb_〉. To address this question, we now evaluate *hb* expression profiles without normalization (Fig. 6b). It is useful to note again that our staining and imaging steps were performed on a side-by-side basis for all the thermal cohorts. In addition, our *hb* FISH intensity data were only adjusted by an embryo-specific background subtraction, a procedure documented to preserve a linear relationship between intensity and *hb* mRNA level^22^ (see Methods). Fig. 6b shows significant divergences of the un-normalized *hb* expression profiles, particular the expression levels, in embryos maturing at different temperatures. To quantify the *hb* expression level, we measured un-normalized FISH intensity values at the plateau region, *hb*_plat_, for individual embryos^22^. Fig. 6c shows the mean values of such measurements: 〈*hb*_plat_〉 = 11.9 ± 4.3, 11.2 ± 3.9, 8.6 ± 3.5, and 18.1 ± 4.7, for embryos maturing at 18°C, 22°C, 25 °C and 29 °C, respectively (*p* = 0.034, 0.021, and 1.9 × 10^−7^ for 18 °C, 22 °C and 29 °C, respectively, in comparisons with 25 °C). These results show that, unlike the mean *hb* expression boundary position 〈*ξ*_hb_〉 (Fig. 3), its mean expression level at the plateau region 〈*hb*_plat_〉 exhibits a significant sensitivity to temperature despite the fact that all embryos had undergone a double screening procedure for developmental age determination (Fig. 1b). These results further underscore the complex nature of the impact of temperature on a developmental system (see Discussion for further details).

## Discussion

Understanding how the environmental factor of temperature affects molecular reactions relevant developmental decisions can deepen our appreciation and enhance our knowledge of developmental robustness. Here we analyze a relatively “simple” developmental system at a time when the exponential concentration gradient of Bcd is formed in the blastoderm embryo and acts as a transcriptional activator of its target gene *hb*. The “simplicity” of this system, combined with our quantitative tools and the specific design of our study, allow us to extract quantitative insights into how the earliest molecular and developmental events are impacted by temperature. Our results show that, even for this simple developmental system investigated under a carefully designed scheme, the effect of temperature is complex. The fact that both molecular reactions and morphological progression are sensitive to temperature suggests that developmental robustness requires various molecular decisions to be harmonized in both space and time. Our quantifications of the effects of temperature provide an elaboration of the types of specific challenges that an embryo must face and resolve when progressing toward its normal outcome.

Our study provides quantitative evaluations of the effects of temperature on both the exponential shape and amplitude of the Bcd gradient in the embryo. We devised our experimental scheme (Fig. 1a) to allow us to investigate exclusively the effect of temperature on the molecular events taking place in the embryo, as opposed to those taking place during oogenesis. Unlike what was suggested previously^9^, our results show that the amplitude of the Bcd gradient profile exhibits a higher degree of sensitivity to temperature than its length constant (Fig. 2). Under the framework of an idealized SDD model, the steady state morphogen concentration *B* is an exponential function, 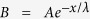, where *A* is the amplitude and *λ* is the length constant. In this model, the length constant is determined by the morphogen protein’s diffusion constant *D* and decay rate constant ω, *λ* = (*D*/ω)^1/2^. Under this framework, we can use effective parameters, *D*_eff_ and ω_eff_, to evaluate Bcd gradient properties in the embryo and the impact of temperature. Our finding that the length constant is largely insensitive to temperature suggests that both *D*_eff_ and ω_eff_ are impacted by temperature in a “coordinated” manner within the temperature range tested.

Our results show that the amplitude of the Bcd gradient profile is sensitive to temperature. Relative to embryos maturing at the “standard” temperature 25 °C, those maturing at either higher or lower temperatures have Bcd gradient profiles with a higher amplitude. According to the idealized SDD model, the amplitude of a concentration gradient *A* is dependent on the production rate *J* in addition to *D* and ω, *A* = *J*/(*D*ω)^1/2^. Using effective parameters under this framework, the amplitude of Bcd gradient is the ratio of *J*_eff_ to (*D*_eff_ω_eff_)^1/2^. If we assume that all these three effective parameters are impacted by temperature in the same direction (i.e., either increase or decrease in their values relative to 25 °C) but by a different degree (i.e., relative changes of the values), a higher amplitude can result from either a preferential increase in *J*_eff_ over (*D*_eff_ω_eff_)^1/2^, or a preferential decrease in (*D*_eff_ω_eff_)^1/2^ over *J*_eff_. A future challenge is to experimentally dissect how these effective parameters may be impacted individually by temperature in a developmental system.

One of the important findings of our current study is that, despite the amplitude differences of the Bcd gradient profiles in embryos maturing at different temperatures, the expression boundary of its target gene *hb* remains largely unaffected by temperature (Figs 3 and 4b). Importantly, we found that the mean Bcd concentration at the *hb* boundary position, 〈*B*_ξhb_〉, remains similar in embryos maturing at different temperatures except 18 °C (Fig. 4a). We note that 18 °C has the largest deviation from 25 °C (in absolute terms) among all the temperatures tested in our current study. Our results thus support a suggestion that, within an “acceptable” temperature range, Bcd activates *hb* transcription when its nuclear concentration crosses a common threshold. These results add to a series of recent studies that have probed the robustness of a concentration-dependent action of Bcd in inducing *hb* expression in the embryo when temperature is not a variable^14^,^18^,^20^,^21^,^22^,^23^,^24^,^27^,^32^,^41^,^48^. They also contrast with previous suggestions that temperature changes can make the local Bcd concentration largely irrelevant to *hb* expression boundary formation^9^.

Before we discuss further the impact of temperature on the observed Bcd-*hb* relationship, it is necessary to introduce several properties of *hb* expression at 25 °C. First, unlike other gap genes whose expression boundaries undergo significant movements along the AP axis as a function of time during nc14, *hb* expression boundary remains relatively stable^35^,^36^,^39^. Second, the *hb* expression boundary is a montage of two overlapping boundaries that have distinct temporal dynamics^29^,^49^,^50^. In particular, as Bcd-activated *hb* transcription undergoes a quick shutdown at early nc14, *hb* transcription at parasegment 4 (PS4) begins to emerge to “reinforce” the *hb* expression boundary^29^. These two properties lead to a *hb* boundary montage that has a relatively stable position but an increasing steepness over time^36^,^47^,^49^,^51^. Thus, the observed divergences of Hill 〈*n*〉 in embryos maturing at different temperatures could be explained by either an alteration of molecular interactions of Bcd molecules in inducing *hb* expression, or differences in the “real ages” of the embryos (see below).

An important aspect of our experimental design is that the embryos used in our analysis had undergone a double screening process according to morphological and molecular standards (Fig. 1). Considering the highly dynamic nature of *hb* transcription in an embryo that is also progressing in morphological terms^29^, it is not unreasonable to question whether these standards themselves are indeed perfectly scaled with time or with one another at different temperatures. We currently favor the possibility that various molecular and morphological “clocks”, i.e., the progressions of different molecular and morphological events, can be impacted by temperature in a manner that is not perfectly harmonized or scaled at a fine resolution. This suggestion highlights the complex nature of the impact of temperature on a developmental system. It also provides a reasonable (but only partial) explanation for the divergences of the observed 〈*n*〉 and 〈*hb*_plat_〉 in embryos maturing at different temperatures. It raises a fundamental question about how various molecular and morphological clocks “negotiate” and “re-align” so that the embryo can proceed further in its “normal” course of development. Given how complex and far-reaching the impact of temperature on a developmental system can be, we ought to have become all that more appreciative of the two hypothetical embryos managing to arrive at hatching when developing at two different temperatures.

## Methods

### Embryo collection scheme

Adults of *w*^*1118*^ were raised under standard conditions at 25 °C. The females were allowed to lay eggs on grape juice agar plates seeded with yeast paste at 25 °C for one hour, at which point the adults and embryos were separated. The embryos were then moved to their respective maturation temperatures, 18 °C, 22 °C, 25 °C and 29 °C for an average of 4, 3, 2, and 1 hour(s), respectively. To accommodate partially the fluctuations in developmental rates and the egg-laying times within a thermal cohort, embryos were collected every fifteen minutes for half an hour before and after the set incubation times (i.e., maturation time at a given temperature has a range of ± 0.5 hr extending from the mean). The collected embryos were then fixed according to standard methods^14^,^22^,^27^.

### Antibody staining and fluorescence *in situ* hybridization

Fixed whole-mount embryos were used in a procedure that combines immuno-staining and mRNA FISH^22^. In our study, immuno-staining was performed using rabbit polyclonal anti-Bcd (1:400, Santa Cruz Biotechnology) and goat anti-rabbit AlexaFluor 594 (1:400, Invitrogen) as the primary and secondary antibodies, respectively. Procedures for the FISH detection of *hb* mRNA^23^ were performed using digoxigenin-labeled RNA probe, detected by mouse anti-digoxigenin (Roche) and goat anti-mouse AlexaFluor 488 (Invitrogen) as the primary and secondary antibodies, respectively. All stained embryos were counterstained with DAPI. To minimize physical deformations, stained embryos were mounted in ProLong Gold (Fisher) using a bridged coverslip as previously described^27^.

Imaging was performed on a Zeiss Imager Z1 ApoTome microscope using a Zeiss Plan 10x Aprochromat objective; image capture was performed using the associated software Axiovision 4.5. The midsagittal section was the target focal plane for each embryo. To allow direct comparisons between different thermal cohorts, we performed staining and imaging steps on a side-by-side basis for all data shown in this study.

### Data Analysis

For data analysis, we first oriented embryo images with the dorsal side facing upwards and the anterior tip to the left. We used two criteria for selecting embryos used in quantitative analysis. First, the images of stained embryos at early nc14 were used to determine the height of the peripheral nucleus along its longitudinal axis. Those embryos that had a nuclear length measuring between 5 and 12 μm were selected in this screening. A second screening was based on the relative level of *hb* at the posterior (Fig. 1b), with those embryos that are within the range of 0.3 to 0.9 selected for data analysis. Measurements of nuclear heights were performed using ImageJ near the middle of the embryo based on the average measured from three nuclei.

Fluorescence intensities for Bcd and *hb* were captured using a digital sliding circle along the nuclear or cytoplasmic layer, respectively, on the dorsal side^14^,^22^,^23^. Our immuno-stained embryos had low background intensities as shown by the raw intensity data (Fig. 1c and S1), and we used the mean values near the posterior for each thermal cohort for a cohort-specific background adjustment. The length constant *Λ* = *λ*/*L* of the Bcd gradient profile of an individual embryo was based on a linear fitting of ln(*B*/*B*_max_) over fractional embryo length *ξ* = *x*/*L* where *B* and *B*_max_ represent background-adjusted Bcd intensity values. For our extracted *hb* mRNA FISH intensity data, we used the average of ten lowest intensity measurements of an embryo for an embryo-specific background subtraction^22^. To obtain normalized FISH intensity profile of an individual embryo, we set the mean of three peak values in the region of *ξ* > 0.4 as 1 (ref. 22).

The approximation of Hill *n* for individual embryos was calculated using normalized *hb* intensity data and Bcd intensity data extracted from locations surrounding the observed *hb* boundary. We used a linear fitting method^47^ of plotting ln[*hb*/(1-*hb*)] against ln(*B*/*B*_ξhb_), where *hb* is the normalized *hb* intensity value at a location, *B* is the corresponding Bcd intensity value, and *B*_ξhb_ is the interpolated *B* value at the calculated *ξ*_hb_ location of the embryo. The linear fit of such a scatter plot represents an estimated Hill *n* necessary to account for the relationship between Bcd and *hb* in the formation of the observed *hb* boundary in the embryo. Statistical analyses were performed using Matlab (R2011b version 7.13) (Mathworks) and the Statistics Toolbox, with individual figure panels generated using Excel (Microsoft).

## Additional Information

**How to cite this article**: Cheung, D. and Ma, J. Probing the impact of temperature on molecular events in a developmental system. *Sci. Rep.* **5**, 13124; doi: 10.1038/srep13124 (2015).

## Supplementary Material

Supplementary Information
