# Supplementary material for: Probing the impact of temperature on molecular events in a developmental system
Source: Sci Rep. 2015 Aug 19;5:13124. doi: 10.1038/srep13124 (PMC4541335; doi:10.1038/srep13124)
Supplement: Supplementary Information [file srep13124-s1.pdf]

<u>**SUPPLEMENTARY INFORMATION**</u>

Probing the impact of temperature on molecular events in a developmental system

David Cheung[1] and Jun Ma[1,2,*]

[1]Division of Biomedical Informatics

[2]Division of Developmental Biology

Cincinnati Children's Research Foundation

3333 Burnet Avenue

Cincinnati, OH 45229, USA

[*]Author for correspondence: Divisions of Biomedical Informatics and Developmental Biology, Cincinnati Children's Hospital Research Foundation, 3333 Burnet Avenue, Cincinnati, OH 45229, USA; 513-636-7977 (phone); jun.ma@cchmc.org (email)

Supplementary Figures

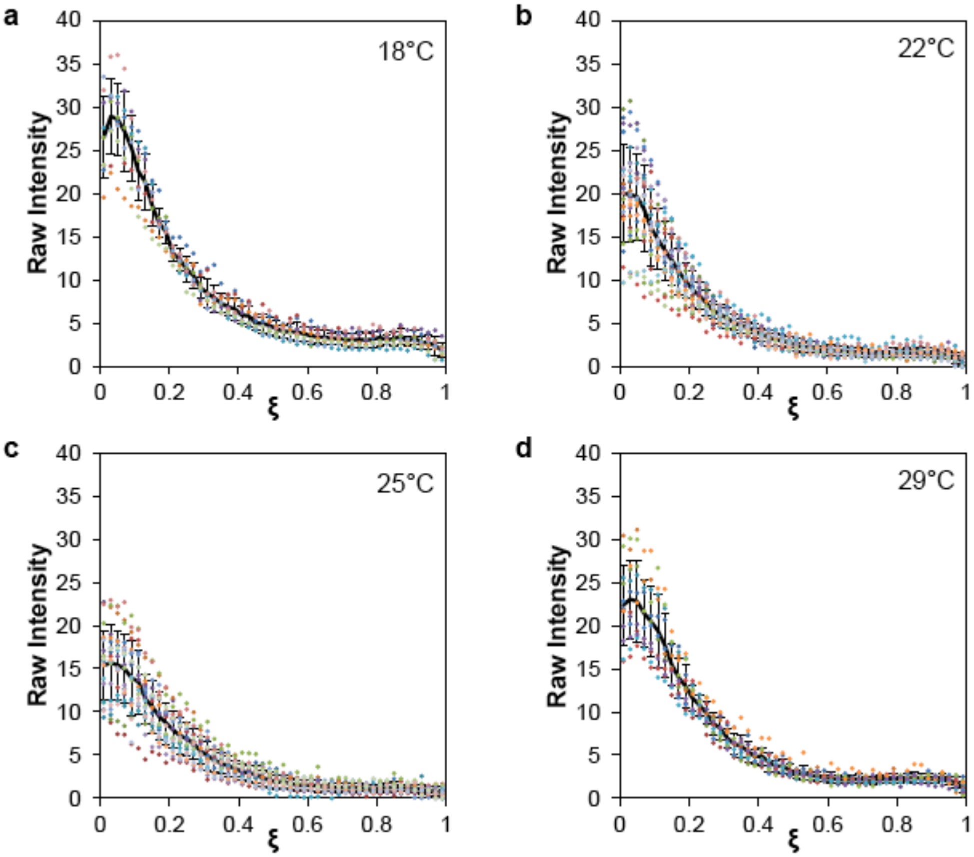

Supplementary Fig. S1. Quantitative measurements of individual Bcd intensity profiles in embryos maturing at different temperatures

a-d) Shown are raw Bcd intensity profiles extracted from individual embryos maturing at the indicated temperatures (18°C, 22°C, 25°C, and 29°C, respectively), plotted as a function of fractional embryo length (ξ). Also shown are the mean and standard deviation (s.d.).

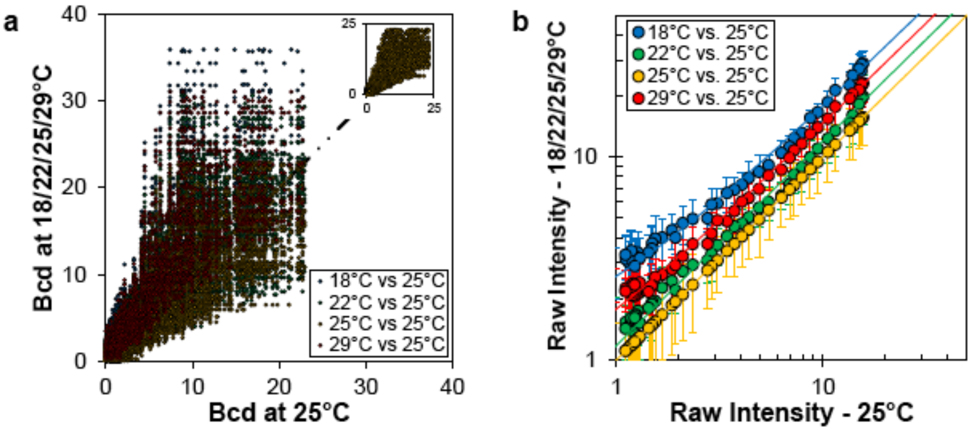

Supplementary Fig. S2. Raw Bcd intensity relationships for embryos raised at different temperatures

a) Shown is a scatter plot of the raw Bcd intensity values from embryos maturing at 18°C, 22°C, 25°C and 29°C plotted against those from embryos at 25°C at their relative positions. The data shown are from all pairwise combinations. The inset shows a scatter plot of intensity values of paired embryos maturing at 25°C. b) Shown are scatter plots of the mean Bcd intensities (and s.d.) between the indicated pairwise combinations of the thermal cohorts. The intensity data are shown in log scale, and a linear fit is shown for each pairwise comparison.

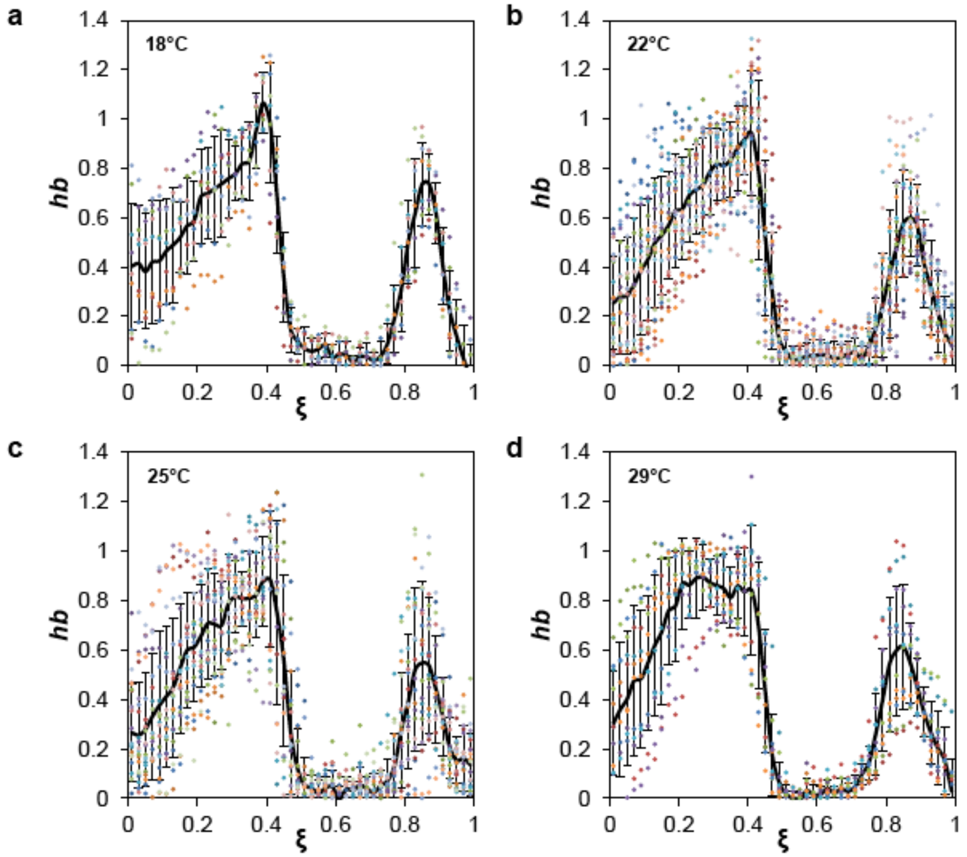

Supplementary Fig. S3. *hb* mRNA FISH intensity profiles from individual embryos maturing at different temperatures

a-d) Shown are normalized *hb* mRNA FISH intensity profiles extracted from individual embryos plotted as a function of fractional embryo length (ξ). The data presented are for each of the indicated thermal cohorts separately. Also shown are the mean s.d.

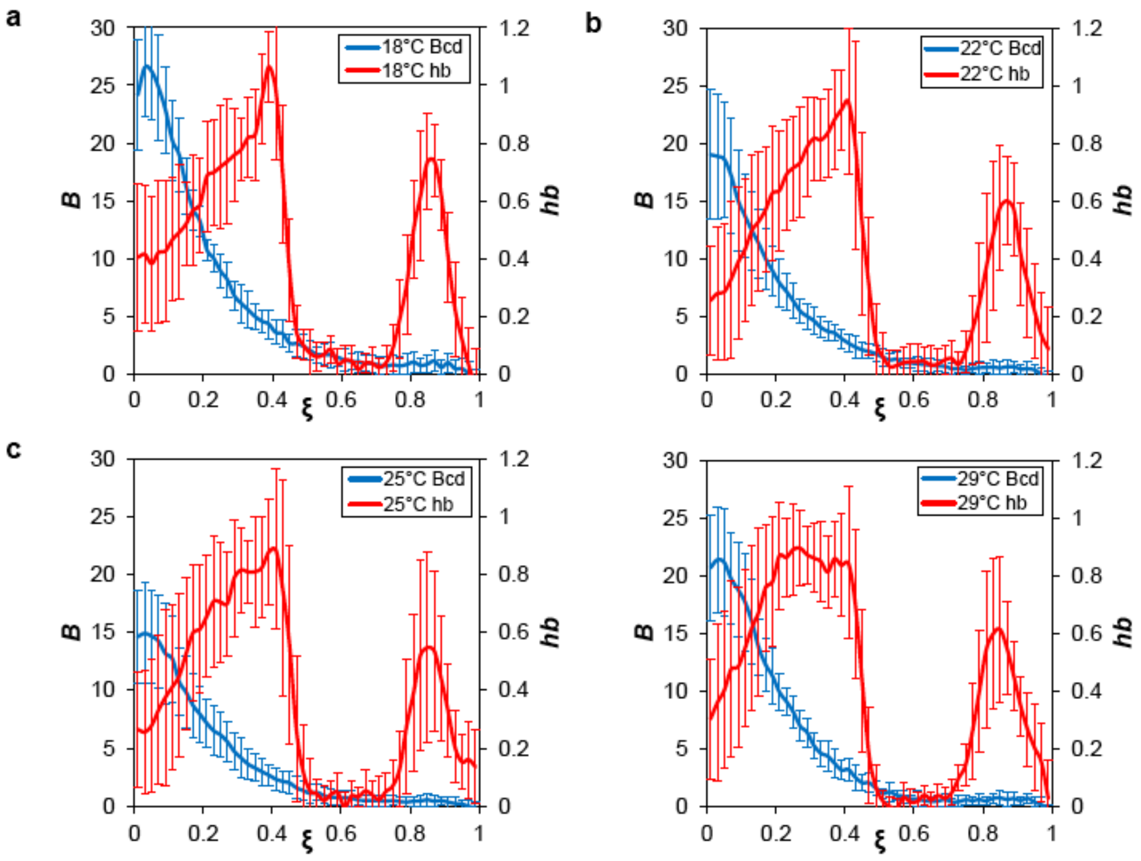

Supplementary Fig. S4. Mean profiles of Bcd and *hb* from embryos maturing at different temperatures

a-d) Shown are superimposed profiles of the mean Bcd intensities (blue, left scale) and the mean normalized *hb* FISH intensities (red, right scale), plotted as a function fractional egg length (ξ). The panels show data for individual thermal cohorts separately. Error bars are s.d.
